# Combining Standard Molecular Typing and Whole Genome Sequencing to Investigate *Pseudomonas aeruginosa* Epidemiology in Intensive Care Units

**DOI:** 10.3389/fpubh.2020.00003

**Published:** 2020-01-28

**Authors:** Bárbara Magalhães, Benoit Valot, Mohamed M. H. Abdelbary, Guy Prod'hom, Gilbert Greub, Laurence Senn, Dominique S. Blanc

**Affiliations:** ^1^Service of Hospital Preventive Medicine, Lausanne University Hospital and University of Lausanne, Lausanne, Switzerland; ^2^Chrono-Environment, Franche-Comté University, Besançon, France; ^3^Institute of Microbiology, Lausanne University Hospital and University of Lausanne, Lausanne, Switzerland

**Keywords:** whole genome sequencing, *Pseudomonas aeruginosa*, molecular typing, molecular epidemiology, double locus sequence typing, genomic epidemiology

## Abstract

*Pseudomonas aeruginosa* is one of the main pathogens responsible for nosocomial infections, particularly in Intensive Care Units (ICUs). Due to the complexity of *P. aeruginosa* ecology, only powerful typing methods can efficiently allow its surveillance and the detection during expanding outbreaks. An increase in *P. aeruginosa* incidence was observed in the ICUs of the Lausanne University Hospital between 2010 and 2014. All clinical and environmental isolates retrieved during this period were typed with Double locus sequence typing (DLST), which detected the presence of three major genotypes: DLST 1–18, DLST 1–21, and DLST 6–7. DLST 1–18 (ST1076) isolates were previously associated with an epidemiologically well-described outbreak in the burn unit. Nevertheless, DLST 1–21 (ST253) and DLST 6–7 (ST17) showed sporadic occurrence with only few cases of possible transmission between patients. Whole genome sequencing (WGS) was used to further investigate the epidemiology of these three major *P. aeruginosa* genotypes in the ICUs. WGS was able to differentiate between outbreak and non-outbreak isolates and confirm suspected epidemiological links. Additionally, whole-genome single nucleotide polymorphisms (SNPs) results considered isolates as closely related for which no epidemiological links were suspected, expanding the epidemiological investigation to unsuspected links. The combination of a first-line molecular typing tool (DLST) with a more discriminatory method (WGS) proved to be an accurate and cost-efficient typing strategy for the investigation of *P. aeruginosa* epidemiology in the ICUs.

## Introduction

*Pseudomonas aeruginosa* is considered one of the main Gram-negative bacteria causing health-care associated infections (1). In the hospital setting, *P. aeruginosa* is widely present in the environment and can be retrieved from different sources, such as respiratory therapy equipment, antiseptics, soap, sinks, and hydrotherapy pools (2). This pathogen was also found to be part of the endogenous microbiota of 2.6–24% of hospitalized patients (3, 4). Patients with compromised host defense mechanisms, such as neutropenia, severe burns, or cystic fibrosis, are particularly affected by this pathogen whose infections lead to high morbidity and mortality (5, 6). *P. aeruginosa* has been previously described as the second most common organism responsible for infections acquired in the intensive care units (ICUs) (7).

*P. aeruginosa* population structure is consensually believed to be panmictic-epidemic (8–10), i.e., a superficially clonal structure with frequent recombination that creates new strains with unique genetic characteristics, in which occasionally highly successful epidemic clones arise. In addition, clinical isolates are indistinguishable from environmental isolates; and there are no specific clones related to a specific habitat (10).

Molecular epidemiological investigations have become essential for active surveillance of infection and detection of outbreaks*. P. aeruginosa* possesses a very complex ecology. For that reason, only powerful typing methods can give insight on the relatedness of strains, and consequently on the routes of colonization and/or infection (11). Pulsed-field gel electrophoresis (PFGE) has been considered the “gold standard” for DNA fingerprinting of *P. aeruginosa* (12–14). However, this method as several disadvantages, such as long analysis time, low intra- and inter-laboratory reproducibility and is labor-intensive, which make it not the optimal method for large investigations (15–17). To overcome these limitations, alternative amplification-based molecular methods have been implemented such as multi-locus sequence typing (MLST), that showed to be efficient in the study of the global population structure of *P. aeruginosa* (18). Another sequence-based method, double locus sequence typing (DLST), based on partial sequencing of two highly variable loci has been successfully used to investigate the epidemiology of *Staphylococcus aureus* and *Pseudomonas aeruginosa* (1, 19–21). With the advance of new technologies, whole genome sequencing (WGS) has been used in recent studies on *P. aeruginosa* evolution and epidemiological investigations in the hospital settings (22–24).

Following an increase in *P. aeruginosa* incidence in the ICUs of the Lausanne University Hospital, clinical and environmental isolates were typed using DLST (1). Three major DLST types were identified, the larger being previously reported as the cause of an outbreak in the burn unit (25). The other two types showed sporadic occurrence with only few cases of possible transmission between patients. The discriminatory power of whole genome sequencing (WGS) was used to further investigate these three major DLST types.

## Materials and Methods

### Bacterial Isolates and Molecular Typing

From 2010 to 2014, *P. aeruginosa* isolates were collected from patients and environment from the five ICUs of the University Hospital of Lausanne. All consecutive patients hospitalized in the ICU with a clinical sample growing *P. aeruginosa* at any site were considered. No routine screening of *P. aeruginosa* carriage was performed. Based on colony morphology, one or several *P. aeruginosa* isolates per clinical sample were chosen for further typing analysis. For patients with prolonged ICU stays, multiple samples were considered for isolate recovery. In 2012, the ICU environment was investigated for the presence of *P. aeruginosa*. Tap water samples and environmental swabs obtained from taps and sink traps of all ICU rooms, as well as from the environment of the hydrotherapy room (including shower trolleys and shower mattresses), were analyzed. Thereafter, sink traps were investigated twice a year.

All isolates were typed by the double locus sequence typing (www.dlst.org) method as previously described (1). Three major DLST types, i.e., types with the highest number of patients, were further analyzed in this study: DLST 1–18 (24 patients), 6–7 (21 patients), and 1–21 (16 patients). For WGS, at least one isolate was selected per patient. If several isolates were collected from one patient, only isolates sampled 15 days apart were selected, unless they belonged from different sample sites. All environmental isolates from the three genotypes (mainly from sink traps) were included. A total of 74 DLST 1–18 isolates (56 clinical and 18 environmental), 50 DLST 6–7 isolates (35 clinical and 15 environmental), and 31 DLST 1–21 isolates (18 clinical and 13 environmental) were selected for WGS. Epidemiologic and genetic data of all clinical and environmental isolates are listed in Table S1.

### Epidemiological Investigation

Epidemiological data (dates of ICU admission and discharge, unit and room of hospitalization and clinical data) were retrieved from the hospital databases and used to construct the timeline of patient's hospital stay and annotate the phylogenetic trees. Epidemiological links between patients or environment were considered in the following situations: (i) patients hospitalized during overlapping periods in the same ICU, or (ii) patients showing an identical DLST type with an environmental sample isolated in the same unit during the period of the study. An outbreak was defined as two or more cases with epidemiological links. A case with no epidemiological links was considered as a single case.

### DNA Extraction and Whole Genome Sequencing

A single colony was picked and incubated into 5 ml Lysogenic Broth (LB) to reach an early exponential phase. We extracted genomic DNA using the GenElute bacterial genomic DNA kit (Sigma-Aldrich, St. Louis, MO, USA). Whole genome sequencing was performed at the Lausanne Genomic Technologies Facility (GTF, University of Lausanne). The sequencing libraries were prepared using the Nextera DNA Library Preparation Kit (Illumina, San Diego, CA, USA) for 100-bp paired-end sequencing runs on Illumina HiSeq 2500, aiming for a 100-fold coverage. All reads data have been deposited with the National Center for Biotechnology Information (https://www.ncbi.nlm.nih.gov/) under the accession project number PRJNA503802.

### SNPs and Phylogenetic Analysis

Isolates' sequence types (ST) were assigned from the short reads data by the Short Read Sequence Typing 2 (SRST2) software (26). A first step of downsampling the raw reads to a given threshold coverage was added. Its purpose was to avoid the occurrence of excessive depth which creates non-informative data and higher computational costs. The depth threshold was set to 70× since this was the minimum read depth observed in our dataset. Complete reference genomes were created by sequencing the first collected clinical isolate of each ST with both PacBio and Illumina HiSeq technologies (27). The subsampled reads were then mapped against their respective complete reference genome with BWA-MEM (https://arxiv.org/abs/1303.3997). Variant calling was performed with FreeBayes (https://arxiv.org/abs/1207.3907) with a minimum mapping quality of 60. Putative phages, repeat regions and potential recombination regions were excluded from the genome alignment. Putative phages were found with PHASTER (28). Repeat regions were detected using an in-house script based on NUCmer (29) with a minimum percentage of identity of 80, and a minimum length of 80 to report a repeat region. Recombination search was performed with an in-house script on the VCF file acquired with FreeBayes (https://arxiv.org/abs/1207.3907) using a probability of 0.001 to remove a region of high SNPs density and a window size of 2000 for SNPs counting.

A maximum likelihood (ML) tree was constructed from the final SNPs alignment using the PhyML algorithm implemented in Seaview version 4.6.1 (30). Tree visualization was done with FigTree version 1.4.3 (http://tree.bio.ed.ac.uk/software/figtree).

### *In silico* Identification of Resistance and Virulence Genes

Acquired antibiotic-resistance and putative virulence genes were identified with SRST2 (26) by mapping the short reads against the ARG-ANNOT (31) and VFDB (32) curated databases, respectively. Additionally, the Antimicrobial Resistance Identification By Assembly (ARIBA) tool (33), which combines mapping/alignment and targeted local assembly of the paired sequencing reads, was applied for detection of antimicrobial resistance genes using the Comprehensive Antibiotic Resistance Database (CARD) (34, 35) as reference.

## Results

### Three DLST Types With Different Epidemiology

**Figure 1:**
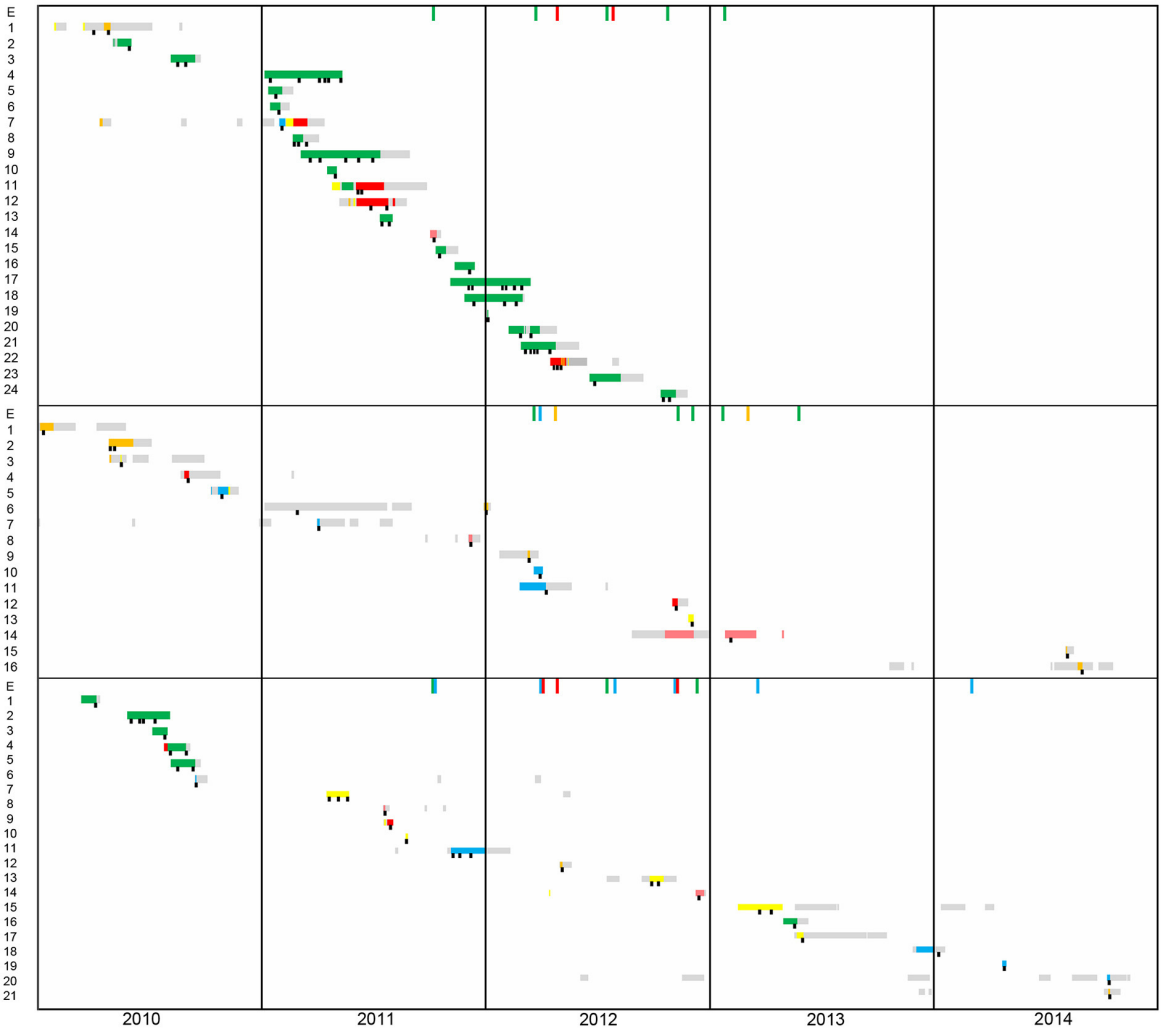
Timeline of patient's hospital stay from 2010 to 2014. The first panel corresponds to patients harboring DLST 1–18 (*N* = 24), the second to DLST 1–21 (*N* = 16), and the third to DLST 6–7 (*N* = 21). Each line represents the patient hospitalization period (yellow, ICU1; blue, ICU2; green, ICU3; orange, ICU4; pink Pediatric-ICU; gray, other wards). A black square represents the time points at which *P. aeruginosa* was isolated.

Epidemiological data of patients and environmental isolates included in this study is schematically represented in Figure 1. DLST 1–18 was previously considered responsible for an outbreak in the burn unit from 2010 to 2012 (25). From the 24 patients harboring this DLST type, 19 were hospitalized in the burn unit (ICU 3), and five in other ICUs. Epidemiological links were found between these patients, either through environmental contamination (especially the hydrotherapy shower room and sink traps) or through patient-to-patient transmission. However, Patient 1 was first isolated in the medical ward and afterwards hospitalized in ICU 5, without obvious epidemiological link with other patients.

Within DLST 1–21, epidemiological links were identified only between two patients hospitalized in ICU 2 and an environmental sample retrieved from a sink trap in the same ICU. The remaining patients were dispersed throughout the six ICUs during the study period (Figure 1), suggesting they were not involved in an outbreak. One patient (Patient 6) was infected in the traumatology ward, before its stay in the ICU.

Regarding DLST 6–7, epidemiological links were found between five patients hospitalized in the burn unit in 2010. For the other 16 patients, no epidemiological link was suspected, as patients were not hospitalized in the same ICU during overlapping periods and had no link with environmental samples. Similarly to DLST 1–21, this DLST type occurred mainly sporadically throughout the study period and was not responsible for a major outbreak.

### Congruence Between DLST and MLST

Although DLST allows inter laboratory comparison of genotypes, the universal standard of multilocus sequence typing (MLST) is still widely used for strain comparison and identification. Therefore, the Illumina HiSeq raw reads were used to identify the STs of each isolate. Results showed that all DLST 1–18 isolates belonged to ST1076, DLST 1–21 to ST253 and DLST 6–7 to ST17, except for one DLST 6–7 isolate, which was found to be of ST845, a single-locus variant from ST17 at the *nuoD* locus. This confirms the previously documented congruence between both methods (36).

### Contribution of WGS to Decipher the Epidemiology of the Three DLST Genotypes

**Figure 2:**
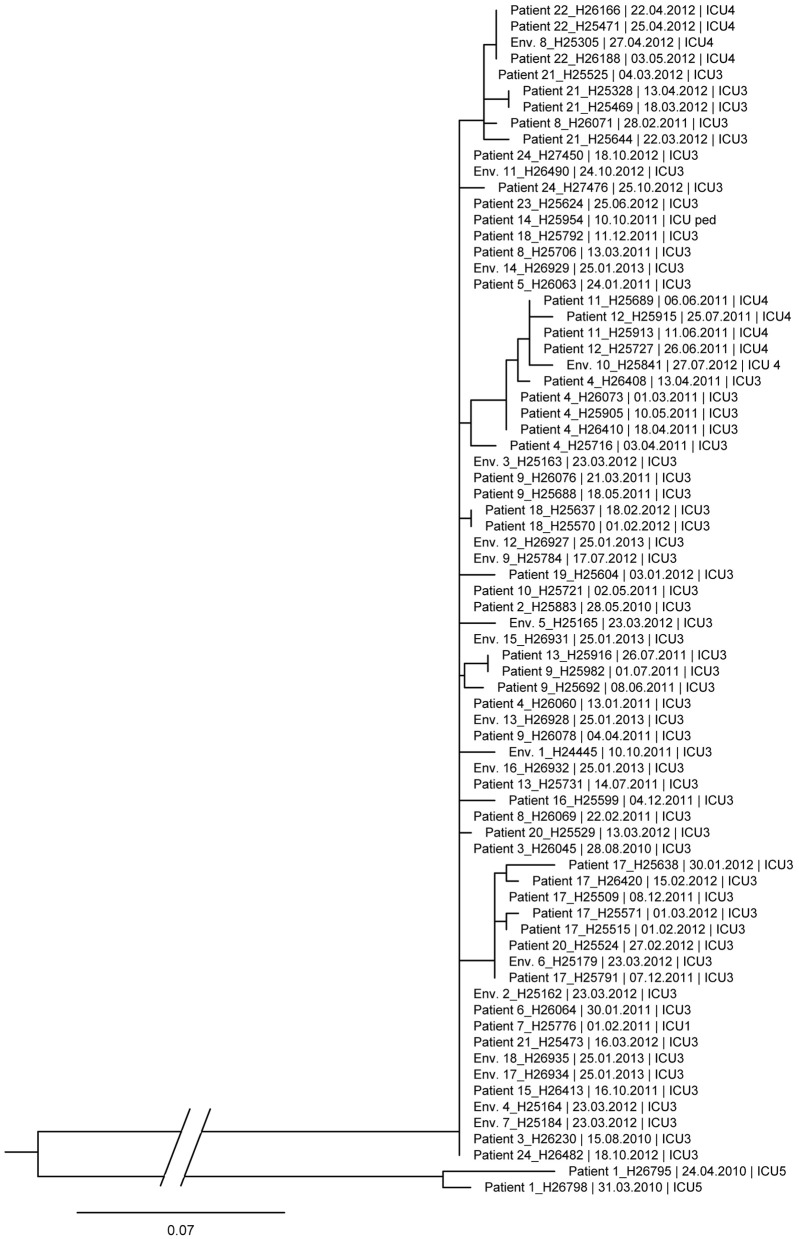
Maximum likelihood tree based on SNP variable sites of all DLST 1–18 isolates. Isolate identification (patient or environment), date and unit of sampling are indicated. Differences of 0–14 SNP were observed between isolates of the outbreak (without Patient 1).

The genetic similarities between isolates of DLST 1–18 are shown in the maximum likelihood tree in Figure 2. Patient 1, who had no epidemiological link with the outbreak, clustered apart from the remaining isolates with 108–120 SNPs differences. Most of the outbreak isolates were closely related with 0–14 SNP differences, confirming the clonal origin of the outbreak. Isolates retrieved from the same patient were different by <10 SNPs, e.g., Patient 4 (0–7 SNPs) and Patient 24 (0–2 SNPs).

**Figure 3:**
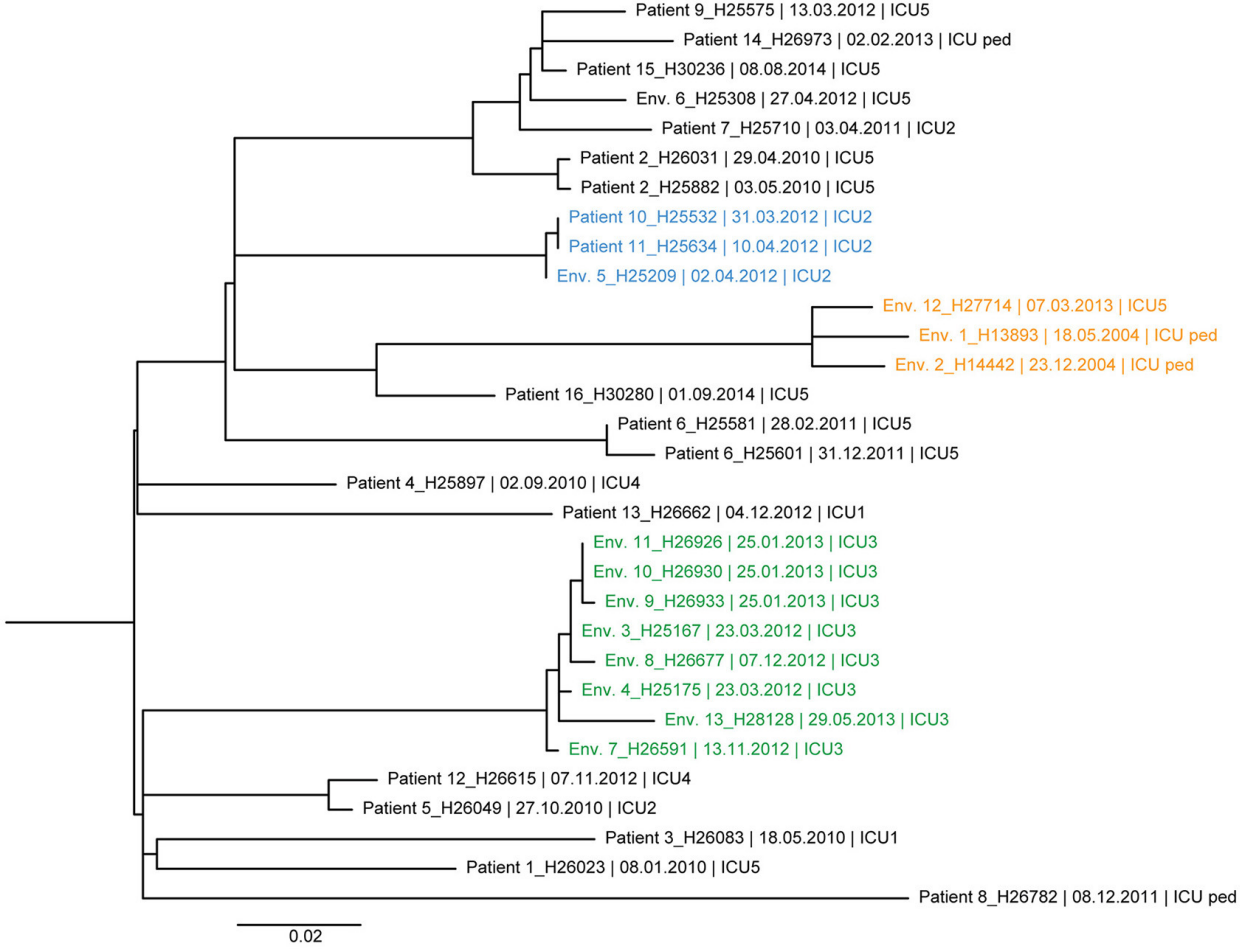
Maximum likelihood tree based on SNP variable sites of all DLST 1–21 isolates. Isolate identification (patient or environment), date and unit of sampling are indicated. Isolates from the suspected outbreak are in blue. The three clades of highly similar isolates from the ICU 2 (0–1 SNP differences), ICU 5/ICU-pediatric (11–14 SNP differences), and burn unit (0–11 SNP differences) are colored in blue, orange, and green, respectively.

**Figure 4:**
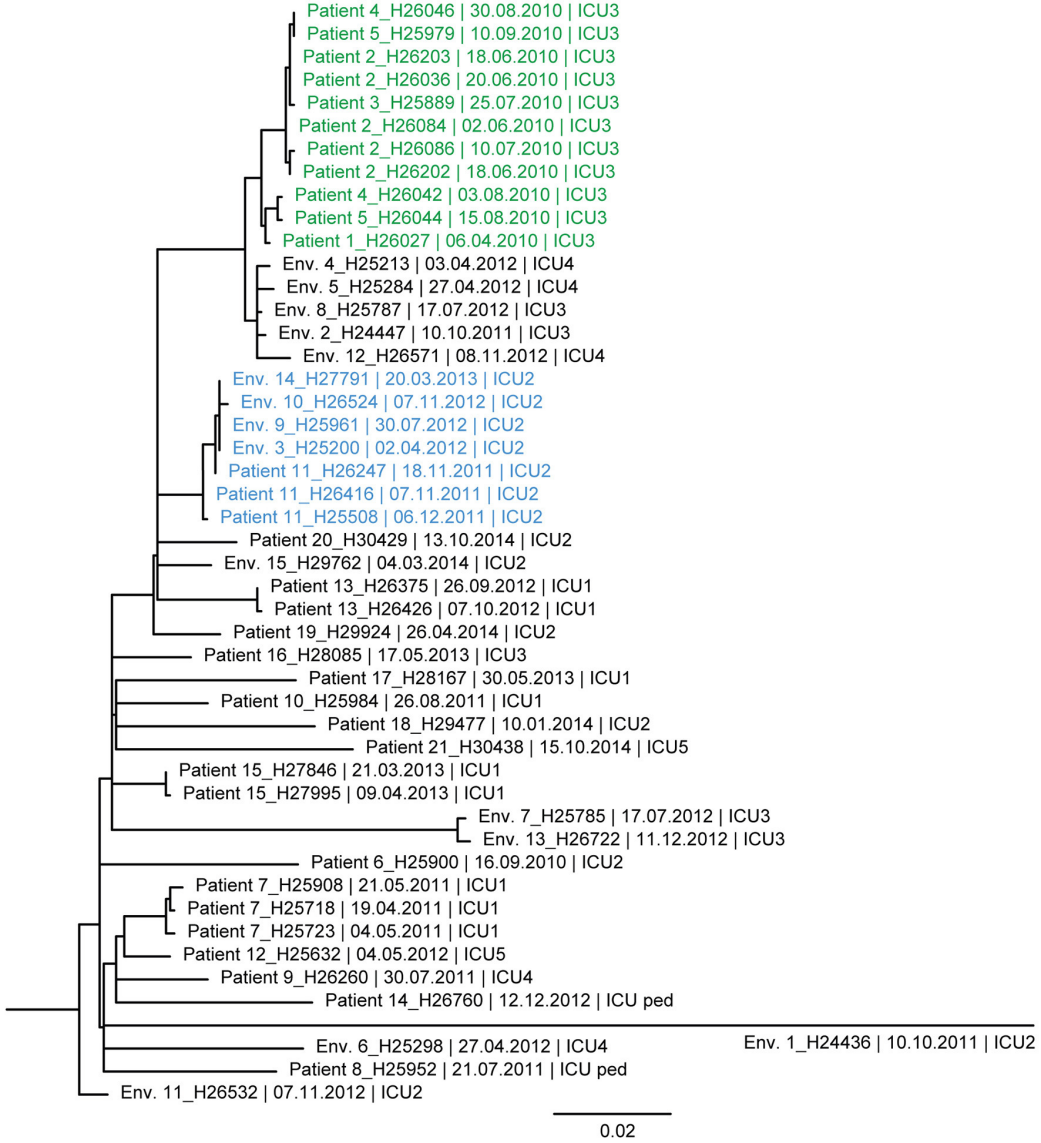
Maximum likelihood tree based on SNP variable sites of all DLST 6–7 isolates. Isolate identification (patient or environment), date and unit of sampling are indicated. A subclade of isolates suspected to be epidemiologically linked in the burn unit is colored in green (0–13 SNP differences). A second subclade, in blue, is composed of isolates from Patient 11 and environmental isolates retrieved in ICU2 (0–7 SNP differences).

The ML trees of DLST 1–21 and 6–7 are shown in Figures 3, 4, respectively. A great diversity was found between isolates of DLST 1–21, reinforcing the premise that they were not all part of a single chain of transmission. Nevertheless, several clades of highly similar isolates were observed. Two isolates from two patients and one isolate from the environment showed 0–1 SNP differences (Patients 10, 11 and Env. 5 in Figure 3), confirming the suspected epidemiological links. Isolates belonging to the same patient were closely related with a maximum of 4 SNPs (Patients 2 and 6). Highly similar isolates were retrieved from the same environment over a 1-year period (sink traps in ICU3), and a 10-year period (pediatric ICU and ICU5 which are adjacent units). Conversely, WGS results also showed high similarities (6 SNP differences) between isolates with no suspected epidemiological link (Patients 5 and 12, hospitalized 2 years apart in two different ICUs).

Similarly, the ML tree of DLST 6–7 showed a large diversity between isolates (Figure 4). Two clades of highly similar isolates were observed. The first was composed of isolates with 0–13 SNPs differences, retrieved from patients with suspected epidemiological links (Patients 1, 2, 3, 4, and 5). The second clade includes isolates from Patient 11 and environmental isolates from the same ICU (0–7 SNPs differences). All isolates recovered from a single patient (Patients 7, 13, and 15) were highly similar with only 0–6 SNPs differences.

### Resistance and Virulence Profiles of *P. aeruginosa* Isolates

The presence/absence of resistance and virulence genes found in the 153 *P. aeruginosa* genomes is listed in Table S2. Although the detected antimicrobial resistance and virulence profiles were very similar among all DLST types, isolates of DLST 1–21 harbored additional genes that were not present in isolates of DLST 1–18 or DLST 6–7. However, specific clusters of closely related isolates observed for each DLST type were not similar in gene content. Several genes encoding resistance to aminoglycosides, fosfomycin, β-lactams, chloramphenicol, polymyxins, bicyclomycin, fluoroquinolones, and inherent multidrug resistance, mainly associated with efflux pump complexes, were found for all isolates (Table S2). A plasmid-encoded ciprofloxacin resistance protein, CrpP, which inactivates the antibiotic by phosphorylation (37), was lacking in all DLST 1–18 isolates but detected in 93% (29/31) and 100% of DLST 1–21 and DLST 6–7 isolates, respectively. The regulatory protein, PvrR, responsible for the conversion between antibiotic-resistant and antibiotic-susceptible forms of *P. aeruginosa* biofilms (38) was only detected in 45% (14/31) of DLST 1–21 isolates. In addition, DLST 1–21 isolates comprised two integron-encoded aminoglycoside adenylyltransferase genes: *aadA6* in 6 isolates from two patients and *aadA7* in 23% (7/31). Resistance to sulfonamides was likewise unique for this genotype due to the presence of the integron-encoded sulfonamide dihydropteroate synthase, *sul1* (39), in 29% (9/31) of the isolates. One isolate belonging to Patient 13 from DLST 6–7 harbored the plasmid-associated *strB* gene, which encodes an aminoglycoside O-phosphotransferase, APH(6)-Id, responsible for streptomycin resistance (40).

Interestingly, *in silico* screening of the presence/absence of putative virulence genes demonstrated that all DLST 1–21 isolates comprising the gene encoding the regulatory protein PvrR, also contained a set of fimbriae genes, *cupD*. These genes are exclusive of the *P. aeruginosa* PA14 strain, which belongs to the same ST253 as the DLST 1–21 isolates, and are regulated by the Rcs/Pvr network (41). As previously suggested by Peña et al. (42), the more frequent type III secretion system (T3SS) exotoxin genes, *exoT* and *exoY*, were common to all DLST types. Nonetheless, the presence of *exoS* was only observed in DLST 6–7 isolates, and ExoU was solely encoded in DLST 1–18 and 1–21 genomes.

## Discussion

In this study, we provide insight into the epidemiology of *P. aeruginosa* in the ICUs of a tertiary care hospital by combining a fast and cheap molecular typing method (DLST) with a more sophisticated and discriminatory method (WGS).

Identifying the isolates' ST by MLST check is one of the advantages of WGS. We were able to determine that DLST 1–21 isolates (ST253) belong to the clinical and international well described clonal complex (CC) PA14, and DLST 6–7 (ST845 and 17) was previously reported as part of the clonal complex C, both CCs being the worldwide most abundant clonal complexes in the *P. aeruginosa* population (43, 44). Little is known so far on ST1076 genomes (DLST 1–18).

The combination of DLST and epidemiological data revealed three genotypes with different epidemiological behaviors. Most of DLST 1–18 patients were hospitalized in the burn unit during overlapping periods. The high number of epidemiological links between patients, along with the wide presence of this DLST type in the environment of the burn unit, previously allowed us to consider this genotype was responsible for an outbreak with an environmental source (25). The discriminatory power of WGS confirmed the high similarity between all DLST 1–18 outbreak isolates (0–10 SNP differences) except for Patient 1. This patient was initially considered as the index case based on DLST, but he was hospitalized in a distant unit without geographical link to the outbreak. According to WGS, this patient could be excluded from the outbreak.

While congruence between epidemiological data and DLST typing was good in this DLST 1–18, this was not the case for the other two DLST types for which only few epidemiological links were recorded. The high diversity between these isolates revealed by WGS confirmed they are not part of a single chain of transmission. Nevertheless, both suspected outbreaks (one in DLST 1–21 and one in DLST 6–7) were confirmed by WGS results and most isolates from a single patient were also found to be highly similar.

The epidemiology of *P. aeruginosa* nosocomial infections is intricate due to the ubiquitous presence of this pathogen in the environment. Since *P. aeruginosa* is capable to survive on wet surfaces such as sinks, sink traps, pipes, and hydrotherapy equipment, several nosocomial outbreaks have been associated with these specific reservoirs. DLST 1–18 environmental isolates retrieved from shower mattresses and sink traps from the hydrotherapy room support the assumption of an environmental source of infection. This assumption was confirmed by results of WGS showing that environmental isolates were highly similar to patients isolates (<10 SNPs).

Apart from contamination in the hydrotherapy room, which was resolved by infection control measures, sink traps were the main reservoir of *P. aeruginosa* in our ICUs during the study period. Our study showed the added value of WGS for the confirmation of epidemiological links between patients and this environment in several occasions. The constant presence of both DLST 1–21 and 6–7 in the environment might have led to sporadic infections, which would explain infections in patients with no epidemiological links (e.g., DLST 1–21, Patients 5 and 12). We also showed that these sink traps of the same or adjacent units can be contaminated with highly similar isolates. More intriguing is the fact that highly similar isolates (<14 SNPs differences) were recovered 10 years apart from sink traps of adjacent units. This low diversity is unexpected considering the long time between isolate sampling, and contrast with isolates retrieved from a single patient, weeks apart, exhibiting already several SNPs differences. One explanation can be the slower evolution of *P. aeruginosa* isolates in the environment of ICUs, as opposed to locations with high selective pressure such as inflamed cystic fibrosis lungs (45).

One limitation of our study relies on the environmental sampling. Extensive sampling was performed in 2012 but not prior to 2012, and only twice a year thereafter. A more frequent and regular sampling might have helped to discover possible epidemiological links between infected patients and environmental sources. It would have also allowed us to thoroughly investigate the diversity of isolates within this ecological niche. In addition, our study focus on the ICU without considering transmission or presence of *P. aeruginosa* in the environment of other hospital units. This limitation is exemplified by one patient infected with one of the major genotypes (Patient 6, DLST 1–21) before its admission to the ICU.

Although we initially hypothesized that DLST 1–18 isolates would have a different resistant/virulent gene content due to its association with a 2.5 years outbreak, the genetic characterization of all genotypes revealed differences primarily between DLST 1–21 and the remaining DLST types (DLST 1–18 and 6–7). Antibiotic resistance in *P. aeruginosa* is commonly associated with the presence of plasmids (46) and several resistance genes are included in mobile genetic elements such as transposons, integron, and IS elements (47–49). The additional resistance observed in DLST 1–21 isolates was associated with plasmid/integron encoded antimicrobial resistance genes. Nevertheless, these genes were only present in an unpatterned subset of DLST 1–21 isolates and were not representative of the entire DLST 1–21 isolate's collection.

A significantly higher number of virulence genes was detected for DLST 1–21 (ST253) isolates. This large set of additional putative additional virulence factors may represent an increased virulence indiscrimination of this DLST type, which is suspected for other highly virulent strains of the same ST, e.g., PA14 (50). *P. aeruginosa* isolates can be differentiated into cytotoxic or invasive, according to their interaction with epithelial cells. The cytotoxic phenotype, as opposed to invasive isolates, encodes for the ExoU toxin (51). *In silico* virulence analysis revealed this toxin is present in both DLST 1–18 and DLST 1–21 isolates, characterizing these genotypes as potentially cytotoxic. However, the expression of this toxin would have to be confirmed by *in vitro* testing.

Being fast and cheap, DLST is performed as a routine surveillance of *P. aeruginosa* in our ICUs by typing all patient and environmental isolates on a quarterly basis. Only if several patients harbor a similar genotype a field epidemiological investigation is undertaken and WGS performed.

In conclusion, we showed the high value of WGS for the investigation of nosocomial *P. aeruginosa* infections. Although WGS costs are decreasing, its implementation as a routine surveillance method for *P. aeruginosa* still comes at a higher price per isolate than the currently used DLST. Additionally, analysis of WGS data requires a certain level of bioinformatic expertise that is not available in all laboratories (52). Thus, using DLST as a first-line molecular typing tool for surveillance and WGS to solve problematic clusters would culminate in an accurate and cost-efficient typing strategy.

## Data Availability Statement

All datasets generated for this study are included in the article/Supplementary Material.

## Ethics Statement

All procedures performed in this study were in accordance with the requirement of the local ethics committee (Commission cantonale d'éthique de la recherche sur l'être humain, Lausanne, Switzerland).

## Author Contributions

BM is the principal investigator. DB is the principal supervisor. BV and MA are bioinformatician supervisors. GP and GG are microbiologist responsible for clinical analysis. LS is the epidemiologist and infection control supervisor.

### Conflict of Interest

The authors declare that the research was conducted in the absence of any commercial or financial relationships that could be construed as a potential conflict of interest.

## Supplementary Material

Table S1 Epidemiological data of each clinical and environmental isolate.

Table S2 Summary of the detected resistance and virulence genes in the 153 *P. aeruginosa* isolates.
